# Does DNA extraction affect the specificity of a PCR method claiming the specific detectability of a genome-edited plant?

**DOI:** 10.1080/21645698.2024.2423441

**Published:** 2024-11-14

**Authors:** Sophia Edelmann, Christian Savini, Dominik Moor, Jörn Lämke, Kathrin Lieske, Marco Mazzara, Hendrik Emons, Joachim Mankertz, Christopher Weidner

**Affiliations:** a Department Method Standardisation, Reference Laboratories, Resistance to Antibiotics, Federal Office of Consumer Protection and Food Safety (BVL), Berlin, Germany; b European Commission, Joint Research Centre (JRC), Ispra, Italy; c Risk Assessment Division, Federal Food Safety and Veterinary Office (FSVO), Bern, Switzerland; d Chemical and Biological Metrology, Federal Institute of Metrology (METAS), Bern, Switzerland; e European Commission, Joint Research Centre (JRC), Geel, Belgium

**Keywords:** DNA extraction, genome editing, GMO detection, official control, oilseed rape (OSR)

## Abstract

Under current EU legislation, genetically modified organisms (GMOs) and derived food and feed products must be authorized as GM food, feed, or seed and appropriate detection methods must be made available for use in official controls. A Real-Time PCR method has recently been published by Chhalliyil et al. claiming to be specific for the detection and identification of genome-edited oilseed rape (OSR) lines commercialized in North America. In a previous study, we have independently assessed this method in three reference laboratories for sensitivity, specificity, and robustness. We found that the method does not meet all the minimum performance requirements (MPR) for GMO testing in the EU, which contradicts the claims of the method developer. Here we show, in addition to the previously published method assessment study that a modified DNA extraction is not the reason for the contradictory findings and does not affect the specificity of the method. We also discuss the procedures recently proposed by the method developers for interpreting PCR results with high Cq values.

## Introduction

*Brassica napus* or oilseed rape (OSR) is primarily grown for its oil-rich seed and is one of the largest vegetable oil sources in the world. In the context of food and feed production, cooking oil from *B. napus* is of major importance with a worldwide consumption of about 34 million tonnes per year.^1^ The meal produced as a by-product is utilized as animal feed, primarily in poultry and dairy industries.^2^ Due to its economic importance trait development is of major interest, but since *B. napus* is a polyploid (allotetraploid) species, traditional genetic methods like hybridization and random mutation are very inefficient and time consuming. With the discovery and advancement of gene editing technologies, the improvement of *B. napus* traits has become faster and more applicable.^3^ Given that gene-edited OSR will soon enter global food chains, official control laboratories face the challenge of reliably detecting even small sequence modifications such as single nucleotide variants (SNV) and identifying and quantifying corresponding events.

A method, intended to specifically detect a SNV in the AHAS1C gene of oilseed rape (OSR), was described in 2020 by Chhalliyil et al.^4^ Their Real-Time PCR method was designed to detect a single base mutation in the AHAS1C gene OSR. According to the authors, the method should be specific for the genome-edited OSR line 5715 (sulfonylurea (SU) tolerant OSR) and closely related OSR lines that were generated thereof and have the same genotype in AHAS1C (e.g., OSR 40 K). They also claim that the method fulfills all performance requirements for analytical GMO methods according to EU regulations. Recently, we have carefully assessed this method^5^ and have shown that the method does not meet all the minimum performance requirements (MPR) for qualitative PCR methods and therefore is not fit-for-purpose for official controls of genetically modified products in the EU. In February 2022, the authors of the article “A Real-Time Quantitative PCR Method Specific for Detection and Quantification of the First Commercialized Genome-Edited Plant”^4^ published a correction and complemented their original article by describing an approach for data evaluation and specificity assessment.^6^

For the method assessment reported in Weidner et al.^5^ we had used exactly the same experimental conditions for Real-Time PCR analysis as described by the method developer. However, the corresponding DNA extracts were generated with different extraction protocols validated for the ground OSR seeds. The MPR document of the European Network of GMO laboratories (ENGL) describes requirements on the performance of the methods developed for the detection, identification, and/or quantification of GMOs based on PCR, including methods for DNA extraction (i.e., quality and quantity of the DNA), and is the legally binding document for EU legislation. In the frame of applications for the authorization of genetically modified food and feed, applicants have to submit a PCR method including an appropriate method for DNA extraction. These methods are validated by the developer as well as by the European Union Reference Laboratory for Genetically Modified Food and Feed (EURL GMFF) for a matrix that is relevant in the context of the application (usually seeds, grain or flour). In official controls, test items are often composed of mixed ingredients from different species with various levels of processing. Therefore, the extraction protocol often has to be adapted according to the sample composition. Control laboratories have to demonstrate by validation or verification that an adapted method is fit-for-purpose for the respective type of sample matrix.

It is a well-established practice in GMO testing that DNA extraction methods and PCR methods are considered as independent modules of the entire analytical process provided that the quality and quantity of extracted DNA fulfil the MPR requirements. For samples composed of seeds or ground seed powder from GM and non-GM material there are cases in which GM and non-GM DNA molecules are not equally well extracted by a particular extraction method, for example, due to different kernel sizes or different water contents of the GM and non-GM parts.^7,8^ Using different extraction protocols might overcome the problem. However, a systematic comparison of DNA extractability from pure GM- and non-GM matrix materials obtained by different extraction methods is not publicly available to our knowledge. This also holds true for complex and processed food and feed samples.

Further, it should be kept in mind that the DNA extraction methods for GMO analysis have so far been assessed and applied in connection with PCR methods that target large unique DNA sequences. More stringent requirements for DNA quality and quantity may have to be established with respect to PCR methods that are dedicated to the detection of a single nucleotide variant (SNV).

Since the specificity claim for the Real-Time PCR method published by Chhalliyil et al.^4^ deviated from the results reported by reference laboratories in the Federal Office of Consumer Protection and Food Safety (BVL, Germany), the European Commission’s Joint Research Centre (JRC) and the Federal Food Safety and Veterinary Office (FSVO, Switzerland), we investigated the potential influence of DNA extraction procedures on the PCR results.

Furthermore, we are discussing a confirmatory testing strategy proposed by Chhalliyil et al. in their correction^6^ that was published shortly after submission of our manuscript.^5^

## Materials and Methods

### Reference Materials

Certified reference material for rapeseed event 73496 (ERM-BF434b; 100% GMO, ground material from seeds) was obtained from the European Commission’s Joint Research Centre (JRC, Geel, Belgium), certified reference material for rapeseed event Rf2 (AOCS 0711-C3; 100% GMO, DNA solution from leaves) and non-modified rapeseed (AOCS 0304-A2; 0% GMO, ground material from seeds) were from the American Oil Chemists’ Society (AOCS, Urbana, IL, USA). Certified reference materials are endorsed by the producer for stability, quality, quantity, and scope of application according to international standards. Seeds of the Clearfield variety ES Decibel CL were kindly provided from Euralis Saaten GmbH (Norderstedt, DE). Seeds of ES Decibel CL were ground at the BVL and ground material was shared with FSVO. Ground material from the OSR variety 40 K seeds was provided by Cibus US LLC (San Diego, CA, USA).

### DNA Extraction and Characterisation of Extracts

All three reference laboratories performed at least two independent DNA extractions from ERM-BF434b and AOCS 0304-A2. For ES Decibel CL two independent DNA extractions were performed by BVL and FSVO, 40 K (positive material) could only be extracted by BVL and FSVO due to legal reasons. In all reference laboratories DNA extracts were independently generated using a DNeasy Plant Mini Kit (QIAGEN, Hilden, Germany, cat. no. 69104) and purified by passing through an Illustra MicroSpin G-50 column (e.g., VWR International GmbH, Dresden, Germany, cat. no. 27533001). This extraction procedure was performed according to detailed instructions kindly provided by Chhalliyil et al., which were based on the manufacturer’s protocols for the DNeasy Plant Mini Extraction Kit and the Illustra MicroSpin G-50 columns, respectively. Moreover, DNA extraction of the 40 K material, ERM-BF434b, AOCS 0304-A2 and ES Decibel CL was performed in the BVL with the Quick-DNA Plant/Seed Miniprep Kit (Zymo Research Europe GmbH, Freiburg, Germany, cat. no. D6020) as described in Weidner et al..^5^ Parts of these extracts, as well as a part of the certified reference material for Rf2 were purified using Illustra MicroSpin G-50 columns according to manufacturer’s instructions (Table 1). The intake of sample material used for a single extraction was 200 mg (DNeasy) and 150 mg (Zymo), respectively, as recommended by the manufacturers.

**Table 1.** Assessment of quantity and quality of DNA extracts generated with different extraction methods.

Sample name	DNA extraction method	DNA concentration [ng/µL]	DNA per PCR [ng]	Inhibition free when tested with FatA(A) TaqMan Universal Mix	Inhibition free when tested with FatA(A) Kapa Probe Fast Mix	Used with method of Chhalliyil et al.
40 K	Zymo	131	300	yes	yes	Fig. 1a
ES Decibel CL, extract #3	Zymo	120	300	yes	not tested^#^	
ES Decibel CL, extract #3	Zymo + G-50	65	300	not tested^#^	yes	
Non-modified rapeseed, extract #3	Zymo	87	300	yes	yes	
Non-modified rapeseed, extract #3	Zymo + G-50	79	300	no	yes	
73496, extract #3	Zymo	141	300	yes	yes	
73496, extract #3	Zymo + G-50	112	300	yes	yes	
40 K	DNeasy + G-50	24	120	no	yes	Fig. 1b,c
ES Decibel CL, extract #1	DNeasy + G-50	49	245	no	yes	Fig. 1b
ES Decibel CL, extract #2	DNeasy + G-50	82	300	yes	not tested^$^	
Non-modified rapeseed, extract #1	DNeasy + G-50	231	300	yes	yes	
Non-modified rapeseed, extract #2	DNeasy + G-50	232	300	yes	yes	
73496, extract #1	DNeasy + G-50	115	300	no	yes	
73496, extract #2	DNeasy + G-50	66	300	no	yes	
Rf2, extract #1	CTAB^§^	120	300	no	yes	Fig. 1c
Rf2, extract #1	CTAB^§^ + G-50	223	300	yes	yes	

DNeasy = extraction with DNeasy Plant Mini Kit according to Chhalliyil et al. (2020),^4^ Zymo = extraction with Quick-DNA Plant/Seed Miniprep Kit.

**Figure 1.**
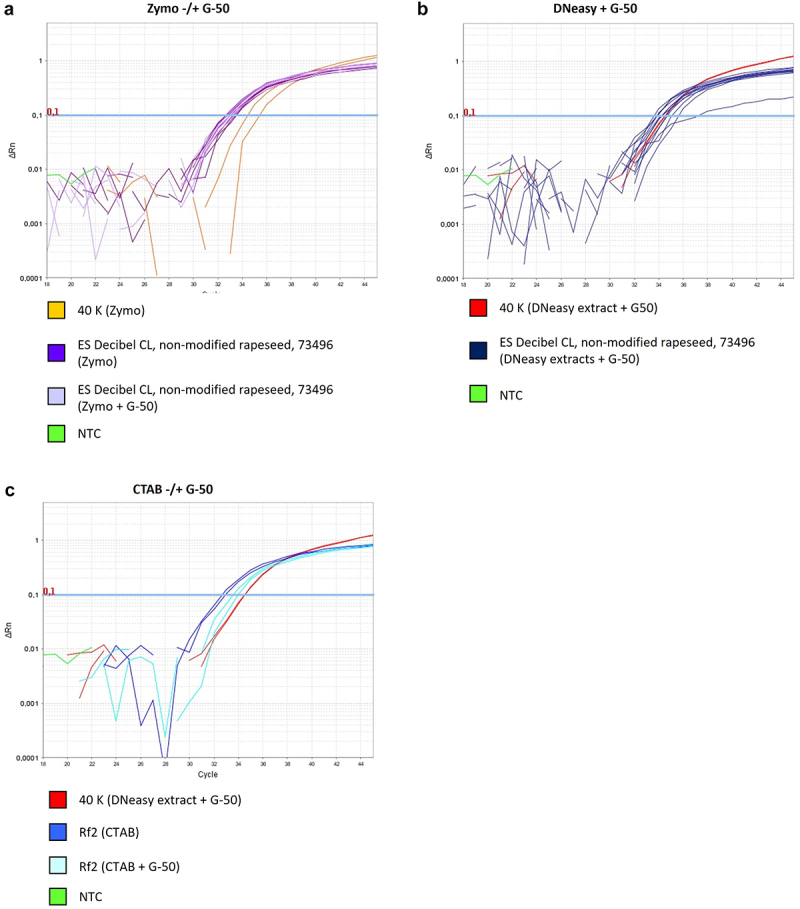
PCR amplification for different rapeseed varieties with the method of Chhalliyil et al.^4^ to detect a SNV using the Kapa Probe Fast Mix on the ABI 7500 Fast System. All extracts (excl. Rf2) were generated in BVL and analyzed in 300 ng total DNA per PCR in duplicate. As positive control, 10 genomic copies of 40 K DNA (equivalent to 0.012 ng total DNA per PCR) were used. A) DNA from ground seed samples was extracted with the Quick-DNA Plant/seed Miniprep Kit (Zymo) and DNA solutions were tested with or without additional G-50 purification. B) DNA from ground seed samples was extracted with DNeasy method as described by Chhalliyil et al. (incl. G-50 purification) C) DNA from Rf2 leaves was extracted with a CTAB method by AOCS and provided extracts were tested without and with additional G-50 purification. NTC, non-template control.

^#^Extract could not be tested due to limited sample volume. An extract, generated with an independent repetition of extraction, was inhibition free when tested with both Master Mixes. With the method of Chhalliyil et al. the extract showed nonspecific amplification with reproducible Cq values as in Fig. 1 and Table S2.

^$^Extract could not be tested due to limited sample volume. An extract, generated with an independent repetition of extraction, was not inhibition free when tested with both Master Mixes. With the method of Chhalliyil et al. the extract showed nonspecific amplification with reproducible Cq values as in Fig. 1 and Table S2.

^§^DNA solution from AOCS.

At the BVL all DNA solutions were quantified by the use of the Quant-iT PicoGreen dsDNA Assay Kit (Cat. no. P7589, Thermo Fisher Scientific, Wilmington, DE, USA) according to the manufacturer’s instructions. Fluorescence was measured with the Infinite F200 Pro (Tecan, Männedorf, Switzerland). At the FSVO DNA concentrations were measured using a NanoDrop One micro-UV/VIS spectrophotometer (Thermo Fisher Scientific, Wilmington, DE, USA). At the EURL GMFF, DNA concentration and the quality of the DNA solutions were assessed considering the UV absorbance at 260 nm and the ratio of UV absorbance at 260 nm and 280 nm with the NanoDrop 1000 Spectrophotometer (Thermo Fisher Scientific, Wilmington, DE, USA), respectively.

### Real‑Time PCR Analysis

Tests were conducted to verify the absence of PCR inhibition for DNA extracted from the study materials according to the ENGL document on method verification.^9^ Therefore, the reference gene FatA(A) method as described in Jacchia et al.^10^ (Table 1) was used in a final volume of 25 µL with 12.5 µL of the validated TaqMan Universal Master Mix for PCR, no AmpErase^TM^ UNG (Cat. no. 4324018, Thermo Fisher Scientific, Wilmington, DE, USA), a DNA dilution series with 300 ng − 1.2 ng DNA per reaction and a PCR programme of 2 min at 50°C, 10 min at 95°C, followed by 45 cycles of 15 s at 95°C and 1 min at 60°C. For one DNA extract with a DNA concentration of 49 ng/µL the maximum sample input was 245 ng instead of 300 ng. Since inhibitory effects had been observed with a number of extracts under these conditions, additional experiments were performed using a reaction mix with the same oligonucleotide sequences and concentrations, the same amounts of DNA, but with the Kapa Probe Fast qPCR Master Mix (Cat. no. KK4703, Sigma-Aldrich, St. Louis, MO, USA) and a slightly adjusted PCR programme of 10 min at 95°C, followed by 45 cycles of 30 s at 95°C and 1 min at 60°C.

The method to detect a SNV in the AHAS1C gene of OSR was conducted according to the protocol of Chhalliyil et al.^4^ (Table S1) in a final volume of 25 µL containing up to 300 ng of a DNA template and 12.5 µL Master Mix (Kapa Probe Fast qPCR Master Mix, Sigma-Aldrich), with final concentrations of 1.6 µM of both primers and 0.8 µM of the probe. The following PCR programme was used: 10 min at 95°C, followed by 45 cycles of 30 s at 95°C and 1 min at 60°C. At the BVL experiments were performed on the ABI 7500 Fast System (Thermo Fisher Scientific), at the FSVO all measurements were done on the Rotor-Gene Q (QIAGEN, Hilden, Germany) and at the JRC (EURL GMFF) PCR experiments were run on the ABI 7500 System (Thermo Fisher Scientific) with heating rates as described previously.^5^

## Results and Discussion

The Real-Time PCR method from Chhalliyil et al. was designed to detect a single base mutation in the AHAS1C gene of OSR.^4^ According to the authors, the method should be specific for the genome-edited oilseed rape carrying this genotype in the AHAS1C gene such as lines 5715 or 40 K. Although the authors claim that the method fulfills all requirements for analytical GMO methods according to EU regulations, we have recently shown in a method assessment^5^ that nonspecific amplification can be detected for several lines of conventional and genetically modified oilseed rape and therefore the MPR criteria are not completely fulfilled. For the current study, the experimental design was extended to investigate whether the method specificity could be influenced by the different DNA extraction procedures used in the studies of Chhalliyil^4^ and of us.^5^ Here, we compared PCR results obtained with DNA extracted by i) a Quick-DNA Plant/Seed Miniprep Kit (abbreviated as Zymo below) as described in Weidner et al.^5^ with and without additional DNA purification using a G-50 MicroSpin column, respectively, or ii) a DNeasy Plant Mini Kit and G-50 MicroSpin column purification with minor protocol modifications as described in Chhalliyil et al..^4^ The DNA concentration of all extracts was quantified and inhibition tests were performed with the FatA(A) method as described in the “Materials and Methods” section above. For extracts that showed inhibition under these validated experimental conditions, additional inhibition tests were performed using the FatA(A) method with the Kapa Probe Fast qPCR Master Mix.

Both DNA extraction protocols resulted in extracts with DNA concentrations above the minimum required to run the PCR protocol (Table 1). However, while DNA extracts obtained with the Quick-DNA Plant/Seed Miniprep Kit were free from inhibition even without additional G-50 column purification, a relevant number of DNeasy extracts purified with G-50 columns, showed inhibition in tests performed with the FatA(A) method using the TaqMan Universal Master Mix. Strikingly, inhibitory effects could be reduced and DNA quality criteria could become compliant with the MPR acceptance criteria by changing the Master Mix for FatA(A) amplification to a Kapa Probe Fast qPCR Master Mix, which was not used in the ring-trial validation for the reference gene detection. The finding that not all DNA extraction protocols are fit-for-purpose to generate DNA solutions without PCR inhibitors for a distinct kind of matrix is well known.^11^ However, our results also show that the occurrence of a PCR inhibition does not only depend on the DNA extraction procedure, but also on the Master Mix of the subsequent PCR method. In fact, four out of seven DNA solutions, extracted from three out of four tested rapeseed lines with the DNeasy/G-50 protocol, caused PCR inhibition when the taxon-specific reference method FatA(A) was used according to the validated protocol with the TaqMan Universal Master Mix. However, no inhibition was observed for the same extracts in tests performed with the FatA(A) method in combination with the Kapa Probe Fast Mix leading to the conclusion that extracts might be of adequate quality when using this experimental design. Because the chemical composition of a PCR Master Mix is an important factor that affects the efficiency of DNA amplification, appropriate additional steps of method validation or verification should be performed when changing a PCR Master Mix of a validated method to assess the risk of nonspecific and false-positive or false-negative results.

Our findings highlight the need to perform appropriate quality controls for DNA extracts and to follow the recommendations described in the ENGL method verification guideline.^9^ In critical cases, it could be useful to go beyond these recommendations and to carry out additional inhibition tests using the same experimental conditions on both the validated GM- and reference gene-specific methods intended for GMO quantification. In case that a full inhibition test would not be possible in a simple format,^9^ two DNA dilutions could be examined with every PCR method (or at least every Master Mix) relevant for the GMO quantification. Such inhibition tests would further contribute to the reliability of the GMO detection results.

It became obvious in the current study that the DNA extraction protocol described by Chhalliyil et al.^4^ resulted in DNA extracts containing inhibitors that could affect the PCR amplification, depending on the PCR Master Mix used. However, a single extraction protocol might not be applicable without modifications for most routine applications used for GMO analysis in food and feed samples. Therefore, when modifying the DNA extraction and/or PCR protocols, any potential impact that may result from the change of the experimental conditions must be assessed. We would generally suggest that only validated DNA extraction methods (validated for the respective type of matrix either by ring trials or by in-house validation) and PCR methods ideally validated with similar extracts should be used. If changes have to be introduced in any of the protocols, there should be a careful method verification. For this reason, in the recent method assessment,^5^ we used a validated DNA extraction method to obtain DNA from OSR varieties and PCR methods validated by the EURL GMFF to ensure that the minimum performance requirements regarding the quantity and quality of the DNA extracts were met. Therefore, the conclusion of the assessment that the original Real-Time PCR method published by Chhalliyil et al.^4^ does not meet all MPR requirements is valid.

We further investigated, if the specificity of a PCR method intended to detect a single nucleotide mutation could be affected by the use of different DNA extraction procedures. Therefore, we generated DNA extracts applying the DNeasy extraction protocol as used by Chhalliyil et al.^4^ and a Quick-DNA Plant/Seed Miniprep Kit protocol (here called Zymo extraction) with and without additional G-50 spin column purification, respectively. We have shown in our previous method assessment^5^ that the published PCR method^4^ is not fully specific for the detection of the SU-tolerant OSR line 40 K, but could lead to nonspecific amplification with other OSR varieties including conventional rapeseed and other GM rapeseed events. Clearfield varieties such as ES Decibel CL are derived from conventional breeding and are therefore non-GM. However, Clearfield varieties have the same target SNV on the AHAS3A gene as the 40 K variety has on the AHAS1C gene of the AHAS multigene family. In the current study, the observation of nonspecific amplification with other varieties than 40 K was verified by additional experiments. For measurements with DNA extracted with the Quick-DNA Plant/Seed Miniprep Kit (Zymo), nonspecific amplification occurred with DNA from non-modified rapeseed, from GM rapeseed events as well as from ES Decibel CL (Fig. 1a). Nonspecific amplification was detected even after additional G-50 spin column purification, and very similar Cq values were obtained for extracts with and without G-50 spin column purification (Fig. 1a, dark and pale purple curves and Supplementary Table S2). For OSR extracts generated with the DNeasy protocol and purified with a G-50 spin column as described in the original method, we also detected nonspecific amplification with distinct amplification curves and Cq values comparable to the positive control (Fig. 1b, Supplementary Table S2). Even for OSR variety Rf2, where the DNA extracts were obtained as certified reference material from AOCS, nonspecific amplification with distinct amplification curves and Cq values comparable to the 40 K positive control were obtained with and without additional G-50 spin column purification (Fig. 1c, pale and dark blue amplification curves, Supplementary Table S2).

This lack of specificity was independently confirmed by all three accredited reference laboratories (Supplementary Tables S3, S4, and Figures S2 and S3). As a consequence, an unequivocal differentiation of SNV-specific and nonspecific signals generated with this PCR method is not possible. For that reason, we have to conclude that the published PCR method^4^ does not meet, even when applied in combination with its originally published DNA extraction method, the minimum performance requirements (MPR) for GMO detection in the EU and is not fit-for-purpose for official control analyses of genetically modified food, feed, or seed.

After being informed of our method assessment^5^ including the observation that nonspecific amplification might be a problem for applying the PCR method as published in Chhalliyil et al.^4^ for official controls, the authors introduced corrections in their original manuscript. In the corrected version of the manuscript,^6^ they suggested that amplification profiles with Cq values < 32 shall be categorized as specific detection, while amplification profiles with Cq values ranging from 32 up to 38 shall be categorized as inconclusive, requiring further investigations to determine the presence of SU-tolerant OSR. The authors presented in the correction a confirmatory testing strategy that should include re-testing of 12 PCR replicates and entails, in the case of 12 positive PCR results, further multiple Sanger sequencings of the amplicon to classify the amplified sequence as positive or negative for the mutation in question. In contrast to the authors’ statement,^6^ the MPR document^12^ is setting the frame for the validation of analytical methods of GMO testing in the EU and does not provide any recommendation for the experimental set-up and interpretation of PCR analyses from routine samples. However, the European Network of GMO Laboratories (ENGL) has developed recommendations for the detection and reporting of unauthorized genetically modified materials.^13^ According to this guidance, a positive analytical result using an event-specific method provides reliable evidence for the presence of a particular unauthorized GMO, provided that the specificity of the method is confirmed by validation studies and that the necessary controls give the expected results.^14^ As we have demonstrated^5^ that nonspecific amplification can be observed by applying the discussed method^4^ even with some wild-type OSR lines, a positive analytical result would not be a reliable evidence for the presence of the targeted SU-tolerant OSR.

The guidance is also stating that a sample, suspected of containing a GMO after screening for common GM elements (e.g., T-nos, *p*-35S), may be considered GM-positive if it has been demonstrated that the positive PCR result has not been caused by the presence of a non-GM donor of the target. However, the SU-tolerant OSR variety of interest does not contain common GM elements and would not give positive PCR results in an element screening approach. In general, genome-edited organisms are not expected to contain common GM elements and consequently, element-based screening approaches would not be applicable.^15^

The proposed strategy of Chhalliyil et al.^6^ to perform subsequent amplicon sequencing of PCR replicates using the AHAS1C-specific primer would be associated with a high probability to generate biased results. For example, if the Real-Time PCR signal has resulted from nonspecific amplification of a non-modified AHAS1C gene in conventional brassica lines with the mismatched primer, the amplicon sequence would be predetermined by the primer sequence. Thus, it would include the single base mutation, regardless of whether the original target DNA contained that specific single base mutation or not. The proposed strategy of amplicon sequencing could be conclusive for the identification of a classical GMO, where the event-specific primer sequence, even when incorporated into the PCR amplicon, is highly specific for a particular GMO and serves as an unequivocal discriminator between the GMO and the wild type material. However, this is not the case for primers designed to discriminate target sequences with only a single nucleotide variation. If sequencing has to be used to detect a single base mutation, a new sequencing method would need to be developed and validated. In general, it seems to be reasonable to choose a sequencing primer that is located about 50 nucleotides upstream of the sequence of interest for a sequencing method intended to confirm the detection of a GMO carrying merely a single base mutation.

By using the PCR method published by Chhalliyil et al.,^4^ we reproducibly observed positive PCR signals with Cq values between 32 and 38 for synthetic DNA templates containing three different AHAS variants that do not contain the mutation of interest as confirmed by Sanger sequencing (Tables S8 – S11 in Weidner et al.^5^). We further found positive PCR signals with Cq values between 32 and 38 for 20 and more replicates from conventional oilseed rape as well as from the genetically modified varieties Rf2, Rf3, and 73496 (Tables S14 – S23 in Weidner et al.^5^). Together, the results lead to the conclusion that the method proposed in Chhalliyil et al.^4^ is not event-specific for 5715 or OSR lines with the same AHAS1C genotype, but would generate false positive results when DNA from conventional and other OSR lines is present. Subsequent Sanger sequencing of the PCR amplicons is not appropriate for the reasons stated above as well as the lack of a validated sequencing method that could reliably determine the single base mutation. Consequently, the method would not be able to distinguish true positive from false positive results.

In conclusion even the corrected method by Chhalliyil et al.^6^ does not meet the EU minimum performance requirements for qualitative PCR methods regarding specificity and robustness and is therefore not fit-for-purpose for official controls of genetically modified products in the EU.

## Supplementary Material

241017 Supplementary Information_revised.docx
