# Supplementary material for: Does DNA extraction affect the specificity of a PCR method claiming the specific detectability of a genome-edited plant?
Source: GM Crops Food. 2024 Nov 14;15(1):352–60. doi: 10.1080/21645698.2024.2423441 (PMC11572162; doi:10.1080/21645698.2024.2423441)
Supplement: 241017 Supplementary Information_revised.docx [file KGMC_A_2423441_SM0287.docx]

**Supplementary Information**

# Does DNA extraction affect the specificity of a PCR Method claiming the specific detectability of a genome-edited plant?

**Author Information**

Sophia Edelmann^1*^, Christian Savini^2^, Dominik Moor^4, 5^, Jörn Lämke^1^, Kathrin Lieske^1^, Marco Mazzara^2^, Hendrik Emons^3^, Joachim Mankertz^1^ and Christopher Weidner^1^

**Affiliations**

^1^ Department Method Standardisation, Reference Laboratories, Resistance to Antibiotics, Federal Office of Consumer Protection and Food Safety (BVL); 10832 Berlin, Germany

^2^ European Commission, Joint Research Centre (JRC), Ispra, Italy

^3^ European Commission, Joint Research Centre (JRC), Geel, Belgium

^4^ Federal Food Safety and Veterinary Office (FSVO), Risk Assessment Division; 3003 Bern, Switzerland

^5^ Federal Institute of Metrology (METAS), Chemical and Biological Metrology; 3003 Bern, Switzerland

***** Correspondence: [sophia.edelmann@bvl.bund.de](mailto:sophia.edelmann@bvl.bund.de)

**Supplementary Tables**

**Table S1**. Sequences and concentrations of oligonucleotides used. [+A] denotes the locked nucleic acid (LNA) modification of desoxyadenosin; 6-FAM, 6-carboxyfluorescein; BHQ1, Black Hole Quencher 1; MGBNFQ, Minor Groove Binder Non-Fluorescent Quencher.

**Table S2.** Cq values for amplification of rapeseed varieties with the SNV method of Chhalliyil et al. (2020) and with the FatA(A) method both using the Kapa Probe Fast Mix on the ABI 7500 Fast System at the BVL. All extracts were analysed with 300 ng total DNA per PCR in duplicate. As positive controls 10 genomic copies of 40 K DNA (equivalent to 0.012 ng total DNA per PCR), either extracted with the Zymo method or the DNeasy method, were amplified.

^§^) DNA solution from AOCS

**Table S3.** Cq values for amplification of rapeseed varieties with the SNV method of Chhalliyil et al. (2020) and with the FatA(A) method both using the Kapa Probe Fast Mix on the ABI 7500 System at the EURL. All extracts were analysed with 300 ng total DNA per PCR in triplicate. As positive controls 10 genomic copies of 40 K DNA (equivalent to 0.012 ng total DNA per PCR) were amplified. DNeasy extracts were independently generated at EURL, Zymo and 40 K extracts were generated at BVL and PCR was performed at EURL.

n.a.) no amplification; *) extracted at BVL; ^§^) DNA solution from AOCS

**Table S4.** Cq values for amplification of rapeseed varieties with the SNV method of Chhalliyil et al. (2020) and with the FatA(A) method both using the Kapa Probe Fast Mix on the Rotor-Gene Q System at the FSVO. All extracts were analysed with 300 ng total DNA per PCR in triplicate. As positive controls 10 genomic copies of 40 K DNA (equivalent to 0.012 ng total DNA per PCR) were amplified. DNeasy extracts were independently generated at FSVO, 40 K extracts were generated at BVL and PCR was performed at FSVO.

n.a.) no amplification; *) extracted at BVL; ^§^) DNA solution from AOCS; ^$^) For FatA(A) determination, 300 ng total DNA were used.

**Supplementary Figures**

**Figure S1.** PCR amplification for different rapeseed varieties with the FatA(A) method using the Kapa Probe Fast Mix on the ABI 7500 Fast System. All extracts (excl. Rf2) were generated in BVL analysed in 300 ng total DNA per PCR in duplicate. As positive control, 10 genomic copies of 40 K DNA (equivalent to 0.012 ng total DNA per PCR) were used. **A**) DNA from ground seed samples was extracted with the Quick-DNA Plant/Seed Miniprep Kit (Zymo) and DNA solutions were tested with or without additional G-50 purification. **B**) DNA from ground seed samples was extracted with DNeasy method as described by Chhalliyil et al. (incl. G-50 purification). **C**) DNA from Rf2 leaves was extracted with a CTAB method by AOCS and provided extracts were tested without and with additional G-50 purification. NTC, non-template control.

**Figure S2.** PCR amplification for different rapeseed varieties with the method of Chhalliyil et al. (2020) to detect a SNV in OSR using the Kapa Probe Fast Mix on the ABI 7500 Fast System at the EURL GMFF. **A**) All extracts (excl. Rf2) were generated at the EURL from ground seed samples using the DNeasy method as described by Chhalliyil et al. (incl. G-50 purification). DNA from Rf2 leaves was extracted with a CTAB method by AOCS and provided extracts were tested without and with additional G-50 purification. For all samples, 300 ng total DNA per PCR (determined by Nanodrop) in duplicate was analysed on a ABI 7500 System. As positive control, 10 genomic copies of 40 K DNA (equivalent to 0.012 ng total DNA per PCR, determined by PicoGreen) were used. **B**) 300 ng DNA (determined by PicoGreen) from extracts generated in BVL (s. Figure 1, Table S2) were independently analysed at the EURL on a ABI 7500 System. As positive control, 10 genomic copies of 40 K DNA (equivalent to 0.012 ng total DNA per PCR) were used. NTC, non-template control.

**Figure S3.** PCR amplification for different rapeseed varieties with the method of Chhalliyil et al. (2020) to detect a SNV in OSR using the Kapa Probe Fast Mix on the Rotor-Gene Q System at FSVO. All extracts (excl. Rf2) were generated at FSVO from ground seed samples using the DNeasy method as described by Chhalliyil et al. (incl. G-50 purification), DNA from Rf2 leaves was extracted with a CTAB method by AOCS and extracts were provided as DNA solutions and analysed without or with additional G-50 purification. For all samples 300 ng DNA (determined by Nanodrop) per PCR in triplicate was analysed on a Rotor-Gene Q System. As positive controls 0.012 ng of 40 K DNA (determined by Nanodrop, equivalent to 10 genomic copies of total DNA per PCR) were used. NTC, non-template control.
